# Twist of Tubular Mechanical Metamaterials Based on Waterbomb Origami

**DOI:** 10.1038/s41598-018-27877-1

**Published:** 2018-06-22

**Authors:** Huijuan Feng, Jiayao Ma, Yan Chen, Zhong You

**Affiliations:** 1 Key Laboratory of Mechanism Theory and Equipment Design of Ministry of Education, Tianjin University, Tianjin, 300072 China; 2 School of Mechanical Engineering, Tianjin University, Tianjin, 300072 China; 3 Department of Engineering Science, University of Oxford, Parks Road, Oxford, OX1 3PJ UK

## Abstract

Origami-inspired mechanical metamaterials have recently drawn increasing attention since their flexible mechanical performance has been greatly enhanced by introducing origami patterns to the thin-shell structures. As a typical origami pattern, the waterbomb tube could be adopted to the design of mechanical metamaterials. However, existing designs predominantly make use of the radial expansion/contraction motion of the structure, thereby limiting its full potential to be explored. Here we report a twist motion of tubular mechanical metamaterials based on waterbomb origami that is previously undiscovered. We demonstrate through a detailed kinematic analysis that the initial twist is a rigid-origami motion if the corresponding row of the tube under twist is fully squeezed with both line and plane symmetry, whereas all the subsequent twist motion requires material deformation. The twist angle per axial strain and its relationship with the geometrical parameters of the tube are revealed. Experimental results show the enhancement in stiffness of the tube with the occurrence of the continuous twist motion. We envisage that this finding could greatly expand the application of the waterbomb tube in the design of origami metamaterials with programmable and tuneable mechanical properties.

## Introduction

Origami, an ancient oriental art of producing 2D or 3D intricate structures through folding a flat sheet of paper, has recently seen surge in a variety of engineering fields. The highlights in the newly formed origami engineering include metamaterials^1–9^, self-folding machine and robots^10–12^, reconfigurable structure^13^, shock-resistance device^14^, packaging^15,16^, and so on. Particularly, the origami-inspired metamaterials refer to the man-made materials that gain their unusual properties from structure rather than composition. Here attention is drawn to the mechanical metamaterials^17^, most of which are based on Miura-ori pattern^1–8^, Resch pattern^3^, and square twist pattern^9^. Among them, the Miura-ori tessellation is the most commonly used. Two folded Miura-based metamaterials were proposed by Schenk and Guest with a negative Poisson’s ratio for in-plane deformations and a positive Poisson’s ratio for out-of-plane bending^1^. Wei *et al*. characterised the geometry and elastic response of a simple periodically folded Miura-ori metamaterial, where in-plane and out-of-plane Poisson’s ratios are equal in magnitude, but opposite in sign, and independent of material properties^2^. Lv *et al*. reported unexceptional coexistence of positive and negative Poisson’s ratio for Miura-based metamaterials once the whole size of the Miura-ori pattern was taken into consideration^3^. A reprogrammable mechanical metamaterial was designed by introducing pop-through defects to the Miura-ori tessellation^4^. Filipov *et al*. assembled rigidly foldable Miura-ori patterns into zipper-coupled tubes that formed reconfigurable cellular metamaterials with enhanced stiffness^5^. Wang *et al*. proposed an in-plane design method for origami-based cylindrical metamaterials with generalised Miura-ori units^6^. Zhou *et al*. presented two new types of origami-inspired mechanical metamaterials with negative Poisson’s ratios and bulk modulus based on the Miura-derivative fold patterns^7^. Recently, Fang *et al*. designed programmable self-locking origami mechanical metamaterials with non-flat-foldable altered Miura-ori tessellations^8^. Meanwhile, the Resch pattern and the square twist pattern are gradually appearing as construction parts for mechanical metamaterials. For example, Lv *et al*. found unusually strong load bearing capability of the mechanical metamaterials based on Resch pattern, which was attributed to the unique way of folding^3^. Silverberg *et al*. studied the bistability characteristics of metamaterials constructed from the square twist pattern^9^. Although the motion of origami structure is utilized in the design of metamaterial to achieve enhanced mechanical property, little work has been done on the kinematic property of the origami pattern itself due to the complexity and multi-degree-of-freedom in the origami motion. One exception is Miura-ori, whose motion is relatively simple and its kinematic analysis has been widely used to reveal the mechanical properties, such as Poisson’s ratio and stiffness^1,6,18^.

Since most engineering materials used to construct origami structures and metamaterials are relatively rigid, a subset of origami that permits continuous motion between folded and unfolded states along the pre-determined creases without stretching or bending of the facets, rigid origami, has drawn special attention. In the mechanism perspective, the creases of rigid origami can be treated as rotation joints and the paper facets treated as links^19,20^. A single-vertex pattern with all creases intersected at the vertex is kinematically a spherical linkage^21^. Then the multi-vertex crease pattern can be modelled as a network of spherical linkages, and its rigidity can be analysed by kinematic approach^22–24^.

Among the vast pool of origami structures, of our particular interest is the waterbomb tube that is made by tessellation of the historically renowned waterbomb bases^25^. Taking advantage of its folding characteristic, the waterbomb tube has not only been one of the favourites for origami artists^26^, but also been adopted in practical applications including a stent graft^27^, a worm robot^28^, and a deformable robot wheel^29^. In all of the above-mentioned applications, the waterbomb tube undergoes only radial expansion/contraction, accompanied by the extending/shortening in the axial direction. A detailed analysis of the radial folding behaviour of the waterbomb tube has been published by the authors^30^, in which a rigorous synchronization of the waterbomb bases along a circumferential row is necessitated, which requires active motion control to realize.

Moreover, playing with a card model of the waterbomb tube reveals that apart from the radial motion, a twist motion also exists starting in the middle of the tube and successively spreading toward both ends, which has not been reported before. Different from the radial motion, the twist of a circumferential row is automatically synchronised, and therefore much easier to generate designed motion and mechanical properties. However, whether the shape change is rigid origami, or whether the facets themselves have to deform, is still unknown. Inspired by a recent work on three-dimensional mechanical metamaterials with twists per axial strain exceeding 2°/%^31^, this waterbomb tube with twist motion will also be used in the design of tubular mechanical metamaterials. Therefore, in this article we embark on a mission to uncover the twist motion behaviour of the waterbomb tube through a detailed kinematic analysis and seek its corresponding mechanical properties caused by the twist motion.

## Results

### Geometry and kinematic setup

**Figure 1:**
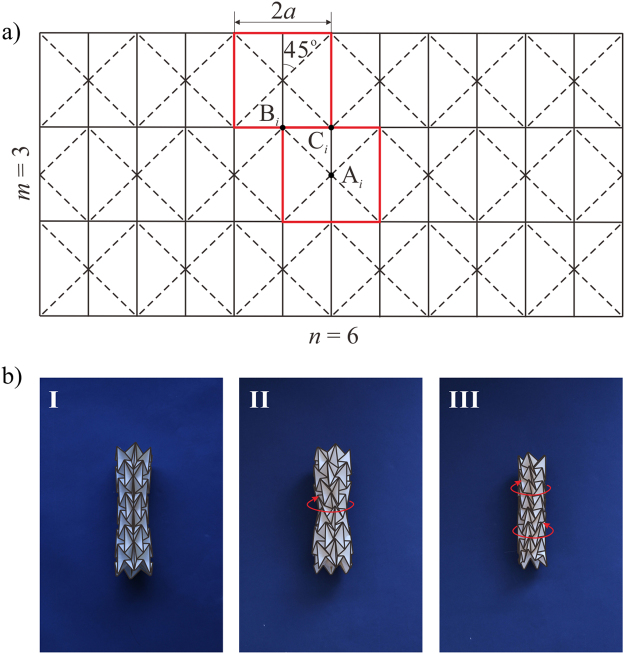
The waterbomb tube. (**a**) The crease pattern formed by tessellating the waterbomb bases in which solid and dash lines are mountain and valley folds respectively. (**b**) Card model of a waterbomb tube with *n* = 6 and *m* = 7 where a twist motion starts from the fully squeezed row and then spreads row to row till the ends of the tube.

The crease pattern of a waterbomb tube is obtained by tessellating the six-crease waterbomb bases, shown in Fig. 1a, where *a* is the half-width of the base, *m* and *n* are the number of bases in the vertical and horizontal direction, respectively. There are three different types of vertices marked by black dots, A_*i*_, B_*i*_ and C_*i*_, where *i* is the row number that the waterbomb base locates. When the two vertical sides of the pattern are joined together, a waterbomb tube is obtained. Playing with the card model of the waterbomb tube, it is found that after the tube reaches its most compact radially contracted configuration (diagram I in Fig. 1b), a further axial compression generates a twist motion. The twist motion occurs from the fully squeezed row, where the largest triangular facets of adjacent waterbomb bases coincide and all the vertices A_0_ at the middle row meet at a single point on the axis of the waterbomb tube (diagram II in Fig. 1b) and then spreads from the middle row toward the rows at both ends of the tube (diagram III in Fig. 1b). To explore the kinematic property of the twist motion, three assumptions of symmetry are made in the subsequent analysis. First, all of the waterbomb bases along the same row behave in an identical manner, and they are placed side-by-side circumferentially. Second, when the twist motion occurs, the twisted base is line-symmetric, i.e., it is rotationally symmetric about a line that passes through the central vertex of the base and is perpendicular to the axis of the tube. Finally, the top and bottom halves of the tube have the same motion behaviour, and the plane that divides the tube into two equal halves is termed as the equatorial plane of the tube.

**Figure 2:**
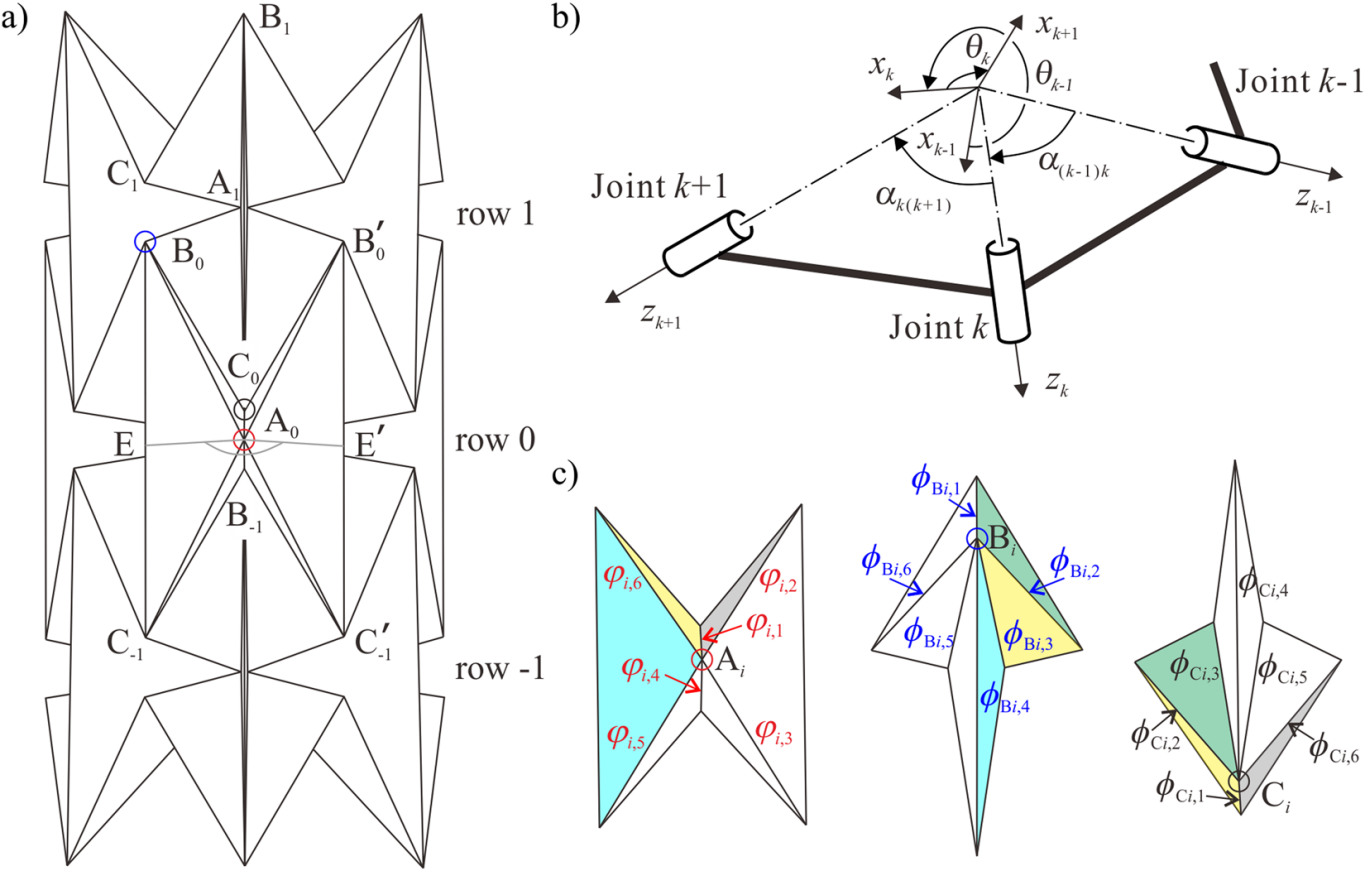
Kinematic setup of a waterbomb tube. (**a**) 3D view of the waterbomb tube when the middle of the tube is fully squeezed. Three types of representative vertices are marked by circles. (**b**) The D-H notations of a portion of a spherical linkage. (**c**) Three types of spherical 6*R* linkages at vertices A_*i*_, B_*i*_ and C_*i*_, where the same color indicates identical sheets.

According to the kinematic equivalence between rigid origami and spherical linkages, the motion around each vertex of the waterbomb tube (Fig. 2a) can be modelled as a spherical 6*R* linkage, then the tube becomes a network of such linkages, which can be analysed with the matrix method in kinematics with the Denavit and Hartenberg notations^32^, see Fig. 2b. The axis *z*_*k*_ is along crease *k* or revolute joint *k*, *x*_*k*_ is the common normal from *z*_*k*−1_ to *z*_*k*_, and *y*_*k*_ is determined by the right-hand rule. Thus the kinematic geometrical parameter *α*_*k*(*k*+1)_ is defined as the angle between *z*_*k*_ and *z*_*k*+1_, positive along the axis *x*_*k*+1_. The kinematic variable *θ*_*k*_ is defined as the angle of rotation from *x*_*k*_ to *x*_*k*+1_ about the axis *z*_*k*_, which measures the rotation between two sheets joined by the crease or revolute joint *k*. In the waterbomb tube, there are three kinds of spherical 6*R* linkages at vertices A_*i*_, B_*i*_ and C_*i*_ (*i* indicates the row number of the base) marked by circles in Fig. 2a and presented in Fig. 2c, which are referred to as linkages **A**_*i*_, **B**_*i*_ and **C**_*i*_ hereafter. The dihedral angles between adjacent sheets connected by the crease are defined as *φ*_*i*,*j*_, *φ*_B*i*,*j*_ and *φ*_C*i*,*j*_ (*j* is increasing in a clockwise sequence with a maximum number equal to 6) for vertices A_*i*_, B_*i*_ and C_*i*_, respectively.

In general kinematics, the closure equation of a spherical 6*R* linkage is

1 $${{\boldsymbol{Q}}}_{21}{{\boldsymbol{Q}}}_{32}{{\boldsymbol{Q}}}_{43}{{\boldsymbol{Q}}}_{54}{{\boldsymbol{Q}}}_{65}{{\boldsymbol{Q}}}_{16}={\boldsymbol{I}},$$

where the rotation matrix ***Q***_(*k*+1)*k*_ is

2 $${{\boldsymbol{Q}}}_{(k+1)k}=[\begin{array}{lll}\cos \,{\theta }_{k} & -\,\cos \,{\alpha }_{k(k+1)}\,\sin \,{\theta }_{k} & \sin \,{\alpha }_{k(k+1)}\,\sin \,{\theta }_{k}\\ \sin \,{\theta }_{k} & \cos \,{\alpha }_{k(k+1)}\,\cos \,{\theta }_{k} & -\,\sin \,{\alpha }_{k(k+1)}\,\cos \,{\theta }_{k}\\ 0 & \sin \,{\alpha }_{k(k+1)} & \cos \,{\alpha }_{k(k+1)}\end{array}],$$

which transforms the expression in the (*k* + 1)th coordinate system to the *k*th coordinate system and ${{\boldsymbol{Q}}}_{k(k+1)}={{\boldsymbol{Q}}}_{(k+1)k}^{-1}$.

Substituting the geometrical parameters of each vertex into the closure equation (1), their kinematic relationships are obtained. Since each crease links two vertices, the dihedral angle on that crease is related to the motion of spherical linkages on both vertices, and the compatibility between neighbouring linkages **A**_*i*_, **B**_*i*_ and **C**_*i*_ yields

3a $${\varphi }_{{\rm{B}}i,3}={{\phi }}_{i,6},\,{\varphi }_{{\rm{C}}i,1}={{\phi }}_{i,1},\,{\varphi }_{{\rm{C}}i,2}={\varphi }_{{\rm{B}}i,2},\,{{\phi }}_{i+1,4}={\varphi }_{{\rm{B}}i,1},\,{{\phi }}_{i+1,3}={\varphi }_{{\rm{C}}i,3},$$

as presented in Fig. 2c, where the sheets with the same color are identical. These relationships hold for the entire waterbomb pattern. At the fully squeezed configuration as shown in Fig. 2a, all the vertices A_0_ at the middle row meet at a single point on the axis of the waterbomb tube, and all points E and E′ in the same row form a circle with point A_0_ as the centre and angle ∠EA_0_E′ as one of the sector angles, where E and E′ are the midpoints of edges B_0_C_−1_ and B′_0_C′_−1_ respectively. Since each waterbomb base in the same row has identical motion,

3b $$\angle {{\rm{EA}}}_{{\rm{0}}}{\rm{E}}^{\prime} =\frac{2\pi }{n}$$

Once these compatibility conditions are satisfied, the motion of the entire tube would be rigid.

### Rigid twist motion of the waterbomb tube

**Figure 3:**
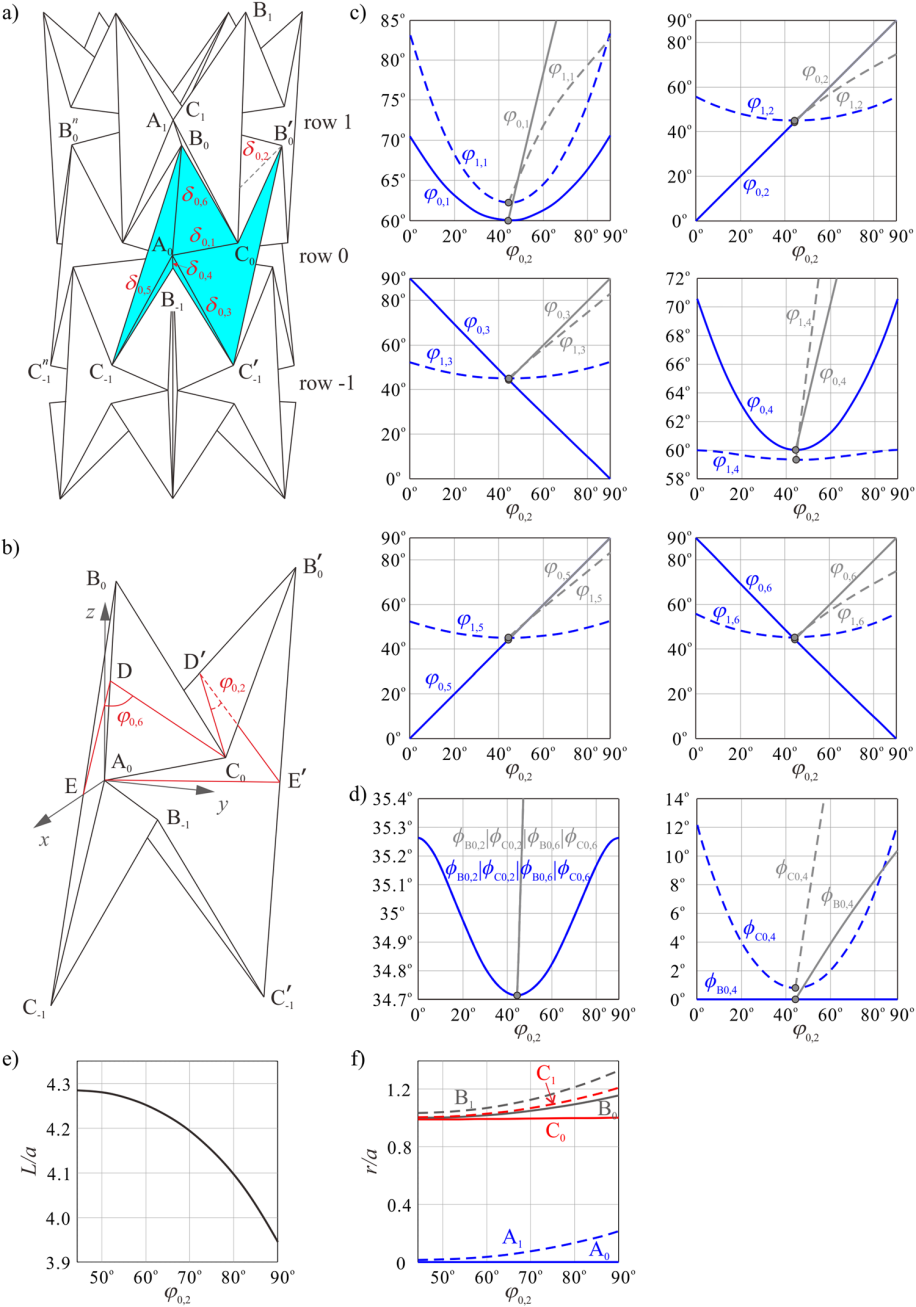
Twist motion on row 0 of a waterbomb tube with *n* = 6 and *m* = 3. (**a**) Partially twisted configuration of the tube. (**b**) Geometry of the line-symmetric linkage **A**_0_ on the twisted row. Kinematic paths of (**c**) linkages **A**_0_ and **A**_1_, and (**d**) linkages **B**_0_ and **C**_0_ in the twist motion (blue) and in the contraction motion (grey). *φ*_0,2_ is taken as input. The bifurcation points are marked by small grey circles. (**e**) Tube length *vs*. *φ*_0,2_. (**f**) Radii of vertices A_*i*_, B_*i*_, and C_*i*_*vs*. *φ*_0,2_.

The card waterbomb tube in Fig. 1b twists from the fully squeezed row with both line and plane symmetry, so we start from this configuration. Here the line symmetry indicates that the upper half of the waterbomb base is in rotational symmetry to the lower half about a line that passes through the central vertex of the base and is perpendicular to the axis of the tube, and the plane symmetry refers to that it is symmetric about a plane formed by two mid mountain creases. Defining the fully squeezed row as row 0, all vertices A_0_ coincide at this configuration, that is, *r*_A0_, the radius of the circle formed by all vertices A_0_ about the axis of the waterbomb tube, becomes 0. Consequently, the dihedral angle *ϕ*_B0,4_ reaches zero. Every crease B_0_C_−1_ is parallel to the axis of the tube. For this instance, the spherical 6*R* linkage at the central vertex A_0_ on row 0 has just completed its motion with both line and plane symmetry, whereas those at the central vertex A_*i*_ on the other rows have only plane symmetry^30^, as shown in Fig. 2a. To facilitate the twist motion, linkage **A**_0_ needs to activate its tilting motion with only line symmetry, see Fig. 3a, where the tube is partially twisted. The geometrical parameters of linkage **A**_0_ are ${\alpha }_{23}^{{{\bf{A}}}_{0}}={\alpha }_{56}^{{{\bf{A}}}_{0}}={90}^{{\rm{o}}}$, ${\alpha }_{12}^{{{\bf{A}}}_{0}}={\alpha }_{34}^{{{\bf{A}}}_{0}}={\alpha }_{45}^{{{\bf{A}}}_{0}}={\alpha }_{61}^{{{\bf{A}}}_{0}}={45}^{{\rm{o}}}$, and the kinematic variables *δ*_0,*j*_(*j* = 1, 2, ..., 6) of the highlighted base in Fig. 3a defined according to the D-H notation have the following relationship

4 $${\delta }_{0,1}={\delta }_{0,4},\,{\delta }_{0,2}={\delta }_{0,5},\,{\delta }_{0,3}={\delta }_{0,6}.$$

By applying equation (4) to the closure equation (1), the following equation can be obtained

5 $$\tan \,{\delta }_{0,1}=\frac{\sqrt{{\rm{2}}}[\sin \,{\delta }_{0,2}+\sin \,{\delta }_{0,3}-\sin ({\delta }_{0,2}+{\delta }_{0,3})]}{2\cos \,{\delta }_{0,2}\,\cos \,{\delta }_{0,3}-\sin \,{\delta }_{0,2}\,\sin \,{\delta }_{0,3}-\cos \,{\delta }_{0,2}-\cos \,{\delta }_{0,3}}.$$

Applying the relationship between the kinematic variables *δ*_0,*j*_ and dihedral angels *φ*_0,*j*_ that *δ*_0,1_ = *π* − *ϕ*_0,1_, *δ*_0,2_ = *π* + *ϕ*_0,2_, *δ*_0,3_ = *π* + *ϕ*_0,3_, *δ*_0,4_ = *π* − *ϕ*_0,4_, *δ*_0,5_ = *π* + *ϕ*_0,5_, *δ*_0,6_ = *π* + *ϕ*_0,6_ to equations (4) and (5), the closure equations of the waterbomb base on row 0 in terms of the dihedral angels, are

6a $${\phi }_{0,4}={\phi }_{0,1},{\phi }_{0,5}={\phi }_{0,2},\,\,{\phi }_{0,6}={\phi }_{0,3},$$

6b $$\tan \,{\phi }_{0,1}=\frac{\sqrt{2}[\sin ({\phi }_{0,2}+{\phi }_{0,3})+\sin \,{\phi }_{0,2}+\sin \,{\phi }_{0,3}]}{2\cos \,{\phi }_{0,2}\,\cos \,{\phi }_{0,3}-\sin \,{\phi }_{0,2}\,\sin \,{\phi }_{0,3}+\cos \,{\phi }_{0,2}+\cos \,{\phi }_{0,3}}.$$

Now let us set up a coordinate system as shown in Fig. 3b with its origin at A_0_, *x* along the direction of $\overrightarrow{{{\rm{A}}}_{0}{\rm{E}}}$, *z* perpendicular to plane EA_0_E′, i.e., the axis of the waterbomb tube, and *y* that is determined by the right-hand rule. Taking points D and D′ as the midpoints of creases A_0_B_0_ and A_0_B′_0_, respectively, we have the following relationship

7 $${\phi }_{0,2}=\angle {{\rm{C}}}_{{\rm{0}}}{\rm{D}}^{\prime} {\rm{E}}^{\prime} \,{\rm{and}}\,{\phi }_{0,6}=\angle {{\rm{C}}}_{{\rm{0}}}{\rm{DE}}.$$

The coordinates of E and E′ are (*a*, 0, 0) and $(a\cos \,\frac{2\pi }{n},\,a\sin \,\frac{2\pi }{n},0)$, respectively, since $\angle {{\rm{EA}}}_{0}{\rm{E}}^{\prime} =2{\pi }/n$. Should the coordinates of B_0_, C_0_ and B′_0_ be denoted by (*x*_B0_, *y*_B0_, *z*_B0_), (*x*_C0_, *y*_C0_, *z*_C0_) and (*x*_B′0_, *y*_B′0_, *z*_B′0_), the following vectors can be obtained

8 $$\begin{array}{rcl}\overrightarrow{{\rm{ED}}} & = & (\frac{{x}_{{\rm{B}}0}}{{\rm{2}}}-a,\,\frac{{y}_{{\rm{B}}0}}{{\rm{2}}},\,\frac{{z}_{{\rm{B}}0}}{{\rm{2}}}),\,\,\overrightarrow{{{\rm{C}}}_{{\rm{0}}}{\rm{D}}}=(\frac{{x}_{{\rm{B}}0}}{{\rm{2}}}-{x}_{{\rm{C}}0},\,\frac{{y}_{{\rm{B}}0}}{{\rm{2}}}-{y}_{{\rm{C}}0},\,\frac{{z}_{{\rm{B}}0}}{{\rm{2}}}-{z}_{{\rm{C}}0}),\\ \overrightarrow{{\rm{E}}^{\prime} {\rm{D}}^{\prime} } & = & (\frac{{x}_{{\rm{B}}^{\prime} 0}}{{\rm{2}}}-a\cos \,\frac{2\pi }{n},\,\frac{{y}_{{\rm{B}}^{\prime} 0}}{{\rm{2}}}-a\sin \,\frac{2\pi }{n},\,\frac{{z}_{{\rm{B}}^{\prime} 0}}{{\rm{2}}}),\\ \overrightarrow{{{\rm{C}}}_{{\rm{0}}}{\rm{D}}^{\prime} } & = & (\frac{{x}_{{\rm{B}}^{\prime} 0}}{{\rm{2}}}-{x}_{{\rm{C}}0},\,\frac{{y}_{{\rm{B}}^{\prime} 0}}{{\rm{2}}}-{y}_{{\rm{C}}0},\,\frac{{z}_{{\rm{B}}^{\prime} 0}}{{\rm{2}}}-{z}_{{\rm{C}}0})\end{array}$$

As $\overline{{{\rm{A}}}_{{\rm{0}}}{{\rm{B}}}_{{\rm{0}}}}\,=\,\overline{{{\rm{A}}}_{{\rm{0}}}{{\rm{B}}^{\prime} }_{{\rm{0}}}}\,=\,\sqrt{2}a,\,\overline{{{\rm{A}}}_{{\rm{0}}}{{\rm{C}}}_{{\rm{0}}}}\,=\,\overline{{{\rm{B}}}_{{\rm{0}}}{{\rm{C}}}_{{\rm{0}}}}\,=\,\overline{{{\rm{B}}^{\prime} }_{{\rm{0}}}{{\rm{C}}}_{{\rm{0}}}}\,=\,a,$ we have

9a $${x}_{{\rm{B0}}}^{2}+{y}_{{\rm{B0}}}^{2}+{z}_{{\rm{B0}}}^{2}=2{a}^{2},\,\,{x}_{{\rm{B}}^{\prime} 0}^{2}+{y}_{{\rm{B}}^{\prime} 0}^{2}+{z}_{{\rm{B}}^{\prime} 0}^{2}=2{a}^{2},\,\,{x}_{{\rm{C0}}}^{2}+{y}_{{\rm{C0}}}^{2}+{z}_{{\rm{C0}}}^{2}={a}^{2},$$

9b $${x}_{{\rm{B}}^{\prime} 0}{x}_{{\rm{C0}}}+{y}_{{\rm{B}}^{\prime} 0}{y}_{{\rm{C0}}}+{z}_{{\rm{B}}^{\prime} 0}{z}_{{\rm{C0}}}={a}^{2},\,\,{x}_{{\rm{B0}}}{x}_{{\rm{C0}}}+{y}_{{\rm{B0}}}{y}_{{\rm{C0}}}+{z}_{{\rm{B0}}}{z}_{{\rm{C0}}}={a}^{2}.$$

Additionally, since $\overline{{{\rm{B}}}_{{\rm{0}}}{\rm{E}}}\,=\,\overline{{{\rm{B}}^{\prime} }_{{\rm{0}}}{\rm{E}}^{\prime} }\,=\,a,$ there are

10 $${x}_{{\rm{B}}0}=a\,{\rm{and}}\,\cos \,\frac{2\pi }{n}{x}_{{\rm{B}}^{\prime} 0}+\,\sin \,\frac{2\pi }{n}{y}_{{\rm{B}}^{\prime} 0}=a.$$

According to the line symmetry of the waterbomb base, the relationship between *y* coordinates of B_0_ and B′_0_ is

11 $${y}_{{\rm{B}}0}=({y}_{{\rm{B}}^{\prime} 0}-a\sin \,\frac{2\pi }{n})/\cos \,\frac{2\pi }{n}.$$

Substituting equations (10) and (11) to equation (9) yields

12 $$\begin{array}{lllll}{z}_{{\rm{B0}}} & = & {z}_{{\rm{B}}^{\prime} 0} & = & \sqrt{(-{y}_{{\rm{B}}^{\prime} 0}^{2}+2a\sin \,\frac{2\pi }{n}{y}_{{\rm{B}}^{\prime} 0}+{a}^{2}\cos \,\frac{4\pi }{n})}/\cos \,\frac{2\pi }{n},\\  &  &  &  & [(\cos \,\frac{2\pi }{n}-1){y}_{{\rm{B}}^{\prime} 0}+a\sin \,\frac{2\pi }{n}]{y}_{{\rm{C0}}}=[\sin \,\frac{2\pi }{n}{y}_{{\rm{B}}^{\prime} 0}+a(\cos \,\frac{2\pi }{n}-1)]{x}_{{\rm{C0}}}.\end{array}$$

Combining equations (8–12) and applying the law of cosines give

13 $$\begin{array}{rcl}{x}_{{\rm{C0}}} & = & \frac{a(\cos \,{\phi }_{0,6}+1)}{2},{y}_{{\rm{C0}}}=a(\cos \,{\phi }_{0,2}-\cos \,\frac{2\pi }{n}\,\cos \,{\phi }_{0,6}+1-\cos \,\frac{2\pi }{n})/(2\sin \,\frac{2\pi }{n}),\\ {z}_{{\rm{C0}}} & = & \frac{\sqrt{2}a[-{\cos }^{2}{\phi }_{0,2}-{\cos }^{2}{\phi }_{0,6}+2\cos \,\frac{2\pi }{n}\,\cos \,{\phi }_{0,2}\,\cos \,{\phi }_{0,6}+2(1-\cos \,\frac{2\pi }{n})]}{(\begin{array}{c}4\{(1-\cos \,\frac{2\pi }{n})[-\cos \,\frac{2\pi }{n}({\cos }^{2}{\phi }_{0,2}+{\cos }^{2}{\phi }_{0,6})+2\cos \,{\phi }_{0,2}\,\cos \,{\phi }_{0,6}\\ {+2(1-\cos \frac{2\pi }{n})(\cos {\phi }_{0,2}+\cos {\phi }_{0,6}+1)]\}}^{1/2}\end{array})}.\end{array}$$

Noting $\overline{{{\rm{A}}}_{{\rm{0}}}{{\rm{C}}}_{{\rm{0}}}}=a$, we can now establish the relationship between φ_0,2_ and *φ*_0,6_ as

14 $${\cos }^{4}{\phi }_{0,6}+{\mu }_{1}{\cos }^{3}{\phi }_{0,6}+{\mu }_{2}{\cos }^{2}{\phi }_{0,6}+{\mu }_{3}\,\cos \,{\phi }_{0,6}+{\mu }_{4}=0.$$

where $${\mu }_{1}=4(\cos \,{\phi }_{0,2}+1-\,\cos \,\frac{2\pi }{n})$$,

$${\mu }_{2}=2(-2{\cos }^{2}\frac{2\pi }{n}-4\cos \,\frac{2\pi }{n}+1)\,{\cos }^{2}{\phi }_{0,2}+12(1-\,\cos \,\frac{2\pi }{n})\,\cos \,{\phi }_{0,2}+8({\cos }^{2}\frac{2\pi }{n}-\,\cos \,\frac{2\pi }{n}+1),$$

$${\mu }_{3}=4[{\cos }^{3}{\phi }_{0,2}+3(1-\cos \,\frac{2\pi }{n})\,{\cos }^{2}{\phi }_{0,2}+2({\cos }^{2}\frac{2\pi }{n}-4\cos \,\frac{2\pi }{n}+1)\,\cos \,{\phi }_{0,2}-4\cos \,\frac{2\pi }{n}(1-\cos \,\frac{2\pi }{n})],$$

$${\mu }_{4}={\cos }^{4}{\phi }_{0,2}+4(1-\cos \,\frac{2\pi }{n})\,{\cos }^{3}{\phi }_{0,2}+8({\cos }^{2}\frac{2\pi }{n}-\cos \,\frac{2\pi }{n}+1)\,{\cos }^{2}{\phi }_{0,2}-16\cos \,\frac{2\pi }{n}(1-\cos \,\frac{2\pi }{n})\,\cos \,{\phi }_{0,2}-4(1-\cos \,\frac{2\pi }{n})\,(3\cos \,\frac{2\pi }{n}+1).$$

Since ϕ_B0,4_ = 0, linkage **B**_0_ degenerates to a spherical 4*R* linkage with joint 4 frozen and joints 3 and 5 combining into one joint. So its closure equations are

15 $$\begin{array}{rcl}\tan \,{\varphi }_{{\rm{B0}},2} & = & \frac{\sqrt{2}}{2}\,\tan \,\frac{{\varphi }_{{\rm{B0}},3}+{\varphi }_{{\rm{B0}},5}}{{\rm{2}}},\,\,\tan \,\frac{{\varphi }_{{\rm{B0}},1}}{{\rm{2}}}=\,\sin \,{\varphi }_{{\rm{B0}},2},\\ {\varphi }_{{\rm{B0}},6} & = & {\varphi }_{{\rm{B0}},2},\,\,{\varphi }_{{\rm{B0}},3}={\phi }_{0,3},\,\,{\varphi }_{{\rm{B0}},5}={\phi }_{0,2}.\end{array}$$

Linkage **C**_0_ remains to be a spherical 6*R* linkage and the closure equations are

16a $${\varphi }_{{\rm{C0}},1}={\phi }_{0,1},\,{\varphi }_{{\rm{C0}},2}={\varphi }_{{\rm{C0}},6}={\varphi }_{{\rm{B0}},2},\,{\varphi }_{{\rm{C0}},5}={\varphi }_{{\rm{C0}},3},$$

16b $$\tan \,\frac{{\varphi }_{{\rm{C0}},3}}{{\rm{2}}}=\frac{-\sqrt{2}\cos \,{\varphi }_{{\rm{C0}},2}+\sqrt{{{\rm{2cos}}}^{2}{\varphi }_{{\rm{C0}},2}+4\sin \,{\varphi }_{{\rm{C0}},2}\,\tan \,\frac{{\varphi }_{{\rm{C0}},1}}{{\rm{2}}}}}{{\rm{2sin}}{\varphi }_{{\rm{C0}},2}},$$

16c $$\tan \,\frac{{\varphi }_{{\rm{C0}},4}}{{\rm{2}}}=\frac{\sqrt{2}\cos \,{\varphi }_{{\rm{C0}},2}\,\sin \,{\varphi }_{{\rm{C0}},3}-{\rm{2sin}}{\varphi }_{{\rm{C0}},2}\,\cos \,{\varphi }_{{\rm{C0}},3}}{\cos \,{\varphi }_{{\rm{C0}},2}\,\cos \,{\varphi }_{{\rm{C0}},3}+\sqrt{2}\sin \,{\varphi }_{{\rm{C0}},2}\,\sin \,{\varphi }_{{\rm{C0}},3}+\cos \,{\varphi }_{{\rm{C0}},2}},$$

which reveals that it is plane-symmetric.

Similarly, we can also set up the closure equations of the other vertices. Motions of those linkages on the rest rows are plane-symmetric and their kinematic relationships are, for linkage **A**_1_,

17a $$\begin{array}{rcl}{\phi }_{1,3} & = & {\phi }_{1,5}={\varphi }_{{\rm{C0}},3},\,{\phi }_{1,4}={\varphi }_{{\rm{B0}},1},\,{\phi }_{1,2}={\phi }_{1,6},\\ \tan \,\frac{{\phi }_{1,6}}{{\rm{2}}} & = & \frac{[\begin{array}{c}-\sqrt{2}\cos \,{\phi }_{1,5}-\,\tan \,\frac{{\phi }_{1,4}}{{\rm{2}}}\,\sin \,{\phi }_{1,5}+(\mathrm{2cos2}{\phi }_{1,5}+2{\tan }^{2}\frac{{\phi }_{1,4}}{{\rm{2}}}{\sin }^{2}{\phi }_{1,5}\\ +2\sqrt{2}{\rm{sin2}}{\phi }_{1,5}\,\tan \,\frac{{\phi }_{1,4}}{{\rm{2}}}{)}^{1/2}\end{array}]}{\tan \,\frac{{\phi }_{1,4}}{{\rm{2}}}-\sqrt{2}\sin \,{\phi }_{1,5}+\,\tan \,\frac{{\phi }_{1,4}}{{\rm{2}}}\,\cos \,{\phi }_{1,5}}\\ \tan \,\frac{{\phi }_{1,1}}{{\rm{2}}} & = & \frac{\sqrt{2}\sin \,{\phi }_{1,5}\,\cos \,{\phi }_{1,6}-\sqrt{2}\sin \,{\phi }_{1,6}}{-\sin \,{\phi }_{1,6}\,\sin \,{\phi }_{1,5}-\cos \,{\phi }_{1,6}+\cos \,{\phi }_{1,5}},\end{array},$$

for linkage **B**_*i*_,

17b $$\begin{array}{rcl}{\varphi }_{{\rm{B}}i,3} & = & {\varphi }_{{\rm{B}}i,5}={\phi }_{i,6},\,\,{\varphi }_{{\rm{B}}i,4}={\varphi }_{C(i-1),4},\,{\varphi }_{{\rm{B}}i,6}={\varphi }_{{\rm{B}}i,2},\\ \tan \,{\varphi }_{{\rm{B}}i,2} & = & \frac{\sqrt{2}\sin \,{\varphi }_{{\rm{B}}i,3}-\,\tan \,\frac{{\varphi }_{{\rm{B}}i,4}}{{\rm{2}}}\,\cos \,{\varphi }_{{\rm{B}}i,3}-\,\tan \,\frac{{\varphi }_{{\rm{B}}i,4}}{{\rm{2}}}}{\sqrt{2}\tan \,\frac{{\varphi }_{{\rm{B}}i,4}}{{\rm{2}}}\sin \,{\varphi }_{{\rm{B}}i,3}+{\rm{2cos}}{\varphi }_{{\rm{B}}i,3}},\\ \tan \,\frac{{\varphi }_{{\rm{B}}i,1}}{{\rm{2}}} & = & \frac{\sqrt{2}\sin \,{\varphi }_{{\rm{B}}i,3}\,\cos \,{\varphi }_{{\rm{B}}i,2}-\,\sin \,{\varphi }_{{\rm{B}}i,2}\,\cos \,{\varphi }_{{\rm{B}}i,3}+\,\sin \,{\varphi }_{{\rm{B}}i,2}}{\cos \,{\varphi }_{{\rm{B}}i,3}+1},\end{array}$$

for linkage **C**_*i*_,

17c $$\begin{array}{rcl}{\varphi }_{{\rm{C}}i,1} & = & {\phi }_{i,1},\,{\varphi }_{{\rm{C}}i,2}={\varphi }_{{\rm{C}}i,6}={\varphi }_{{\rm{B}}i,2},\,{\varphi }_{{\rm{C}}i,5}={\varphi }_{{\rm{C}}i,3},\\ \tan \,\frac{{\varphi }_{{\rm{C}}i,3}}{{\rm{2}}} & = & \frac{-\sqrt{2}\cos \,{\varphi }_{{\rm{C}}i,2}+\sqrt{{{\rm{2cos}}}^{2}{\varphi }_{{\rm{C}}i,2}+4\sin \,{\varphi }_{{\rm{C}}i,2}\,\tan \,\frac{{\varphi }_{{\rm{C}}i,1}}{{\rm{2}}}}}{{\rm{2sin}}{\varphi }_{{\rm{C}}i,2}},\\ \tan \,\frac{{\varphi }_{{\rm{C}}i,4}}{{\rm{2}}} & = & \frac{\sqrt{2}\cos \,{\varphi }_{{\rm{C}}i,2}\,\sin \,{\varphi }_{{\rm{C}}i,3}-{\rm{2sin}}{\varphi }_{{\rm{C}}i,2}\,\cos \,{\varphi }_{{\rm{C}}i,3}}{\cos \,{\varphi }_{{\rm{C}}i,2}\,\cos \,{\varphi }_{{\rm{C}}i,3}+\sqrt{2}\sin \,{\varphi }_{{\rm{C}}i,2}\,\sin \,{\varphi }_{{\rm{C}}i,3}+\,\cos \,{\varphi }_{{\rm{C}}i,2}},\end{array}$$

and for linkage **A**_*i*+1_,

17d $$\begin{array}{rcl}{\phi }_{i+1,4} & = & {\varphi }_{{\rm{B}}i,1},\,{\phi }_{i+1,3}={\phi }_{i+1,5}={\varphi }_{{\rm{C}}i,3},\,{\phi }_{i+1,2}={\phi }_{i+1,6},\\ \tan \,\frac{{\phi }_{i+1,6}}{{\rm{2}}} & = & \frac{[\begin{array}{c}-\sqrt{2}\cos \,{\phi }_{i+1,5}-\,\tan \,\frac{{\phi }_{i+1,4}}{{\rm{2}}}\,\sin \,{\phi }_{i+1,5}+(2{\tan }^{2}\frac{{\phi }_{i+1,4}}{{\rm{2}}}{\sin }^{2}{\phi }_{i+1,5}\\ +{\rm{2cos2}}{\phi }_{i+1,5}+2\sqrt{2}{\rm{sin2}}{\phi }_{i+1,5}\,\tan \,\frac{{\phi }_{i+1,4}}{{\rm{2}}}{)}^{1/2}\end{array}]}{\tan \,\frac{{\phi }_{i+1,4}}{{\rm{2}}}-\sqrt{2}\sin \,{\phi }_{i+1,5}+\,\tan \,\frac{{\phi }_{i+1,4}}{{\rm{2}}}\,\cos \,{\phi }_{i+1,5}},\\ \tan \,\frac{{\phi }_{i+1,1}}{{\rm{2}}} & = & \frac{\sqrt{2}\sin \,{\phi }_{i+1,5}\,\cos \,{\phi }_{i+1,6}-\sqrt{2}\sin \,{\phi }_{i+1,6}}{-\,\sin \,{\phi }_{i+1,6}\,\sin \,{\phi }_{i+1,5}-\,\cos \,{\phi }_{i+1,6}+\,\cos \,{\phi }_{i+1,5}},\end{array}$$

where *i* = 1, 2, …, (*m* − 3)/2.

**Figure 4:**
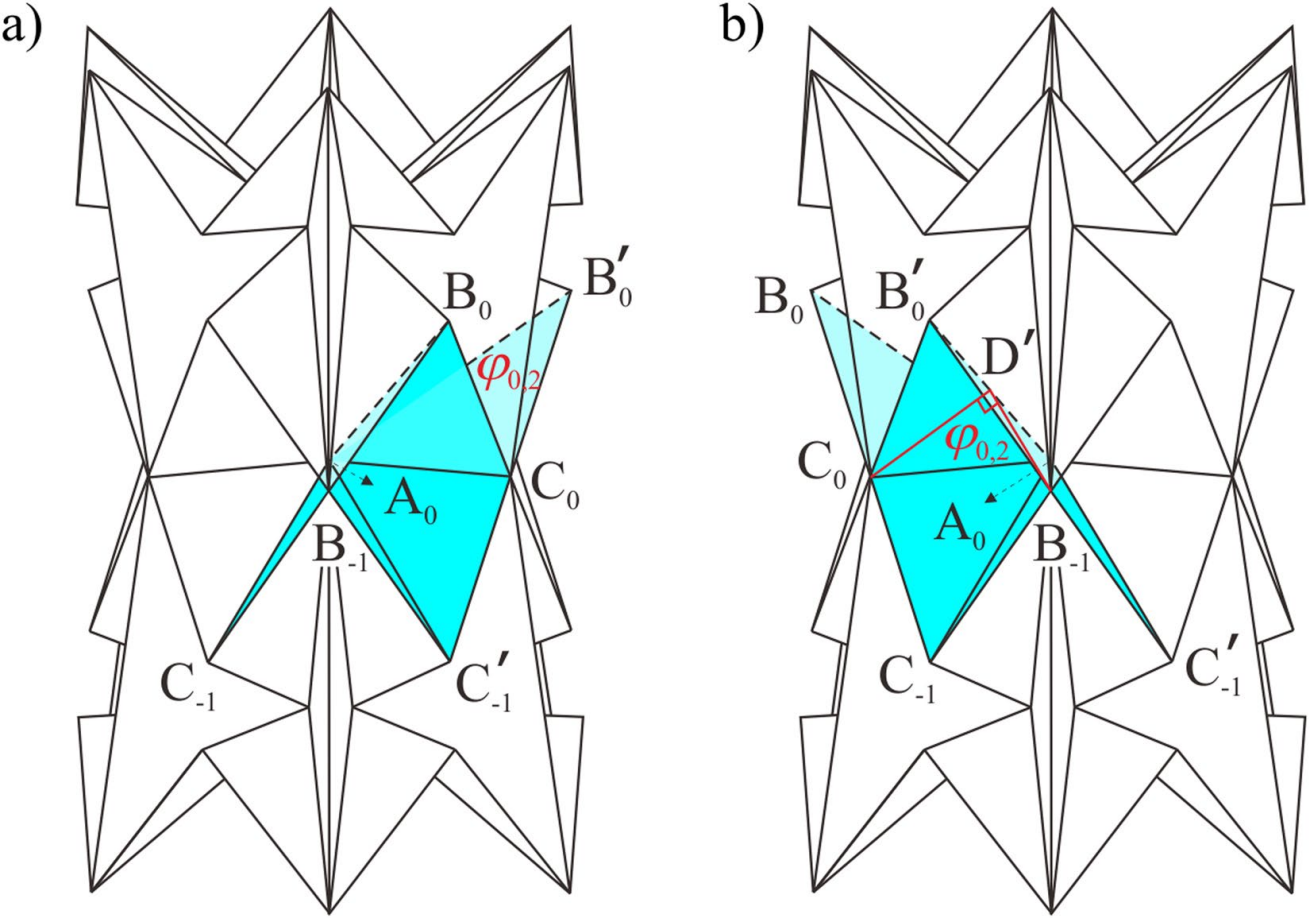
The most compact folding configurations of the waterbomb tube when *n* = 6 and *m* = 3. The two limit positions are obtained where (**a**) *φ*_0,2_ = 0°, and (**b**) *φ*_0,2_ = 90°.

Hence, equations (6) and (14–17) form the kinematic relationship set of the entire tube. Only one variable, *φ*_0,2_, is needed to determine the motion of the tube, i.e., the tube is rigidly foldable with one degree of freedom. The kinematic paths of the tube with *n* = 6 are plotted as in Fig. 3c,d. The range of φ_0,2_ is determined by the two limiting positions: *φ*_0,2_ = 0° and *φ*_0,2_ = 90°, see Fig. 4a,b, which correspond to counter-clockwise and clockwise twist, respectively. In Fig. 3c, the blue lines show the kinematic paths of linkages **A**_**0**_ (in blue solid lines) and **A**_**1**_ (in blue dash lines) in the twist motion, which indicates that linkage **A**_**0**_ embarks on the tilting motion, whereas linkage **A**_1_ on the adjacent row is still in plane-symmetric motion. From the partial kinematic paths of linkages **B**_0_ (in blue solid lines) and **C**_0_ (in blue dash lines) in the twist motion in Fig. 3d, it can be seen that starting from zero, *ϕ*_B0,4_ always remains zero even when linkage **A**_0_ undergoes tilting motion, thereby verifying that linkage **B**_0_ actually degenerates into a spherical 4 *R* linkage. In addition, *ϕ*_C0,4_ is always positive during the twist motion.

Furthermore, the switch from the contraction to the twist motion is, in fact, a motion bifurcation of linkage **A**_0_ from a line- and plane- symmetric motion to a line-symmetric motion. This can be clearly demonstrated by plotting the kinematic paths of the contraction motion^30^ in the same diagrams given in Fig. 3c,d (grey and grey dash lines), in which those bifurcation points are marked by shaded circles. The twist motion further shortens the overall length, *L*, of the tube with *m* = 3 (Fig. 3e), but the radii, *r*, of the vertices become slightly larger (Fig. 3f). It enables all the bases on row 0 to reach its most compact folding configuration at either *φ*_0,2_ = 0° or *φ*_0,2_ = 90° (Fig. 4a,b). Animation of the rigid twist motion of the waterbomb tube with *n* = 6 and *m* = 3 is presented in the Supplementary Video S1.

Having demonstrated from the kinematic analysis that the twist of row 0 is a rigid motion, next we investigate the range of the input kinematic variable *φ*_0,2_. Several circumstances need to be considered. First, the region of *φ*_0,2_ is constrained by two limit positions where *φ*_0,2_ = 0° and *φ*_0,2_ = 90°. Second, all the other rows of the tube are found to expand with the twist motion on row 0 by analyzing their kinematic relationship set. So another limit of *φ*_0,2_ is generated when the linkage **A**_(*m*−1)/2_ on row (*m* − 1)/2 is fully deployed with *φ*_(*m*−1)/2,1_ = 180°. And finally, all the other rows except for the twisted one move with plane symmetry, and interferences of facets should be taken into account when determining the range of the rigid twist motion. *r*_A*i*_ ≥ 0 and *ϕ*_B*i*,4_ ≥ 0 should always be satisfied during the motion, hence the region of φ_0,2_ is further restricted.

With the range of the input kinematic variable *φ*_0,2_, the maximum twist angle between two ends, ${{\rm{B}}}_{0}^{n}{{\rm{B}}}_{0}{{\rm{B}}^{\prime} }_{0}$ and ${{\rm{C}}}_{-1}^{n}{{\rm{C}}}_{-1}{{\rm{C}}^{\prime} }_{-1}$, of row 0 along tube axis in Fig. 3a, *θ*_t_, can be calculated

18 $$\tan \,\frac{{\theta }_{{\rm{t}}}}{2}=\frac{\cos \,{\phi }_{0,2{\rm{\min }}}-\,\cos \,{\phi }_{0,6{\rm{\max }}}}{\tan \,\frac{\pi }{n}(\cos \,{\phi }_{0,2{\rm{\min }}}+\,\cos \,{\phi }_{0,6{\rm{\max }}}+2)},$$

where ${\phi }_{0,2\min }$ is the minimum value of *φ*_0,2_, and ${\phi }_{0,6\max }$ is calculated by equation (14) when *φ*_0,2_ is taken as ${\phi }_{0,2\min }$.

**Figure 5:**
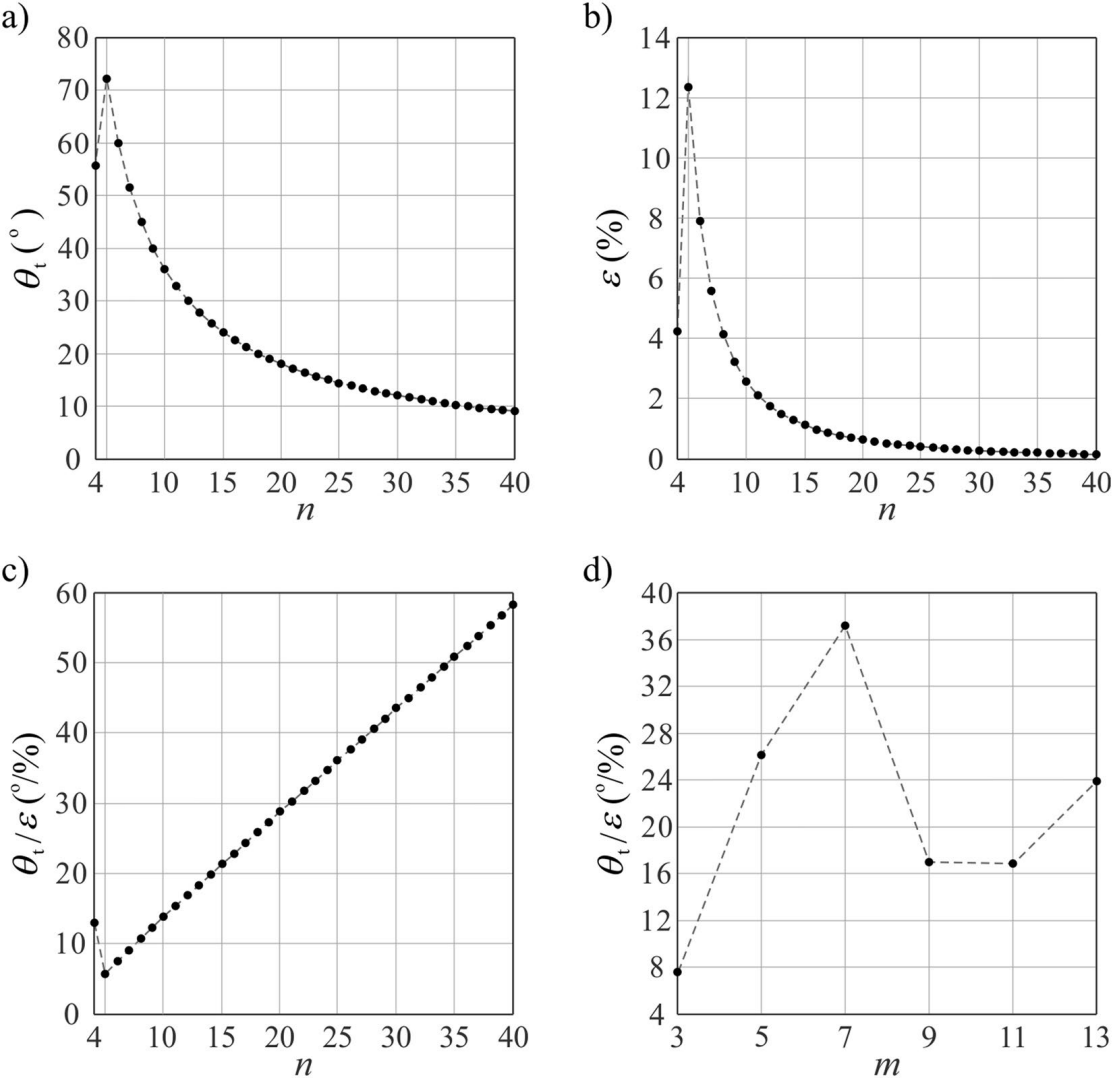
Rigid twist of the waterbomb tube. (**a**) The twist angle between two ends of a tube, *θ*_t_, *vs*. the number of bases in a row, *n*, when the number of rows *m* = 3. (**b**) The axial strain of the tube *ε vs*. *n* when *m* = 3. (**c**) The twist angle per axial strain *θ*_t_/*ε vs*. *n* when *m* = 3. (**d**) *θ*_t_/*ε vs*. *m* when *n* = 6. The twist angle here is calculated as the maximum rigid twist between two ends of a tube, and the axial strain is calculated as the strain when the maximum rigid twist is reached.

Since only row 0 of a tube generates rigid twist motion while all the other rows keep plane symmetry, the twist angle *θ*_t_ between two ends of the tube is independent of the number of rows *m*, while affected only by the number of bases in a row *n*. We take *m* = 3 to demonstrate the relationship between *θ*_t_ and *n*, see Fig. 5a. Here *n* is taken from 4 to 40 since no rigid twist motion exists when *n* < 4. It can be seen that *θ*_t_ increases when *n* increases from 4 to 5. This is due to the fact that when *n* = 4, the twist angle *θ*_t_ is obtained where row 1 is fully expanded with *φ*_1,1_ = 180°. The tube cannot reach the most compact folding configuration with *φ*_0,2_ = 0° (Fig. 4a) as the case of *n* = 5, leading to a smaller twist angle. When *n* surpasses 4, *θ*_t_ monotonically reduces with *n* for the reason that equation (18) degenerates to *θ*_t_ = 360°/*n* in this case. The maximum value of *θ*_t_ is reached when *n* = 5, where *θ*_t_ = 72°.

The rigid twist degree of freedom of the waterbomb tube makes it a suitable candidate for the design of chiral mechanical metamaterials which twist when axially deformed. This property can be characterised by the twist angle per axial strain, *θ*_t_/*ε*^31^. The axial strain, *ε*, considering compression strain as positive, can be calculated as

19 $$\varepsilon =\frac{{L}_{0}-{L}_{t}}{{L}_{0}},$$

where *L*_0_ and *L*_*t*_ are the overall length of the tube at the fully squeezed configuration with *ϕ*_B0,4_ = 0 and at the fully twisted configuration with ${\phi }_{0,2}={\phi }_{0,2\min }$, respectively. They can be calculated as

20 $$L=2{z}_{B((m-1)/2)},$$

where the cylindrical coordinates of each vertex are

21 $$\begin{array}{ccc}{r}_{{\rm{A}}i} & = & a\sin \,\frac{{\varphi }_{{\rm{B}}i,4}}{2}/\sin \,\frac{\pi }{n},\,{r}_{{\rm{B}}i}=a\sin \,\frac{{\phi }_{i,1}}{2}/\sin \,\frac{\pi }{n},\,{r}_{{\rm{C}}i}=a\sin \,\frac{{\phi }_{i+1,4}}{2}/\sin \,\frac{\pi }{n},\\ {z}_{{\rm{B}}i} & = & {z}_{{\rm{A}}i}+\sqrt{2{a}^{2}-{r}_{{\rm{B}}i}^{2}-{r}_{{\rm{A}}i}^{2}+2{r}_{{\rm{A}}i}{r}_{{\rm{B}}i}\,\cos \,\frac{\pi }{n}},\,{z}_{{\rm{C}}i}={z}_{{\rm{A}}i}+\sqrt{{a}^{2}-{({r}_{{\rm{C}}i}-{r}_{{\rm{A}}i})}^{2}},\\ {z}_{A(i+1)} & = & {z}_{{\rm{C}}i}+\sqrt{2{a}^{2}-{r}_{{\rm{C}}i}^{2}-{r}_{A(i+1)}^{2}+2{r}_{{\rm{C}}i}{r}_{A(i+1)}\,\cos \,\frac{\pi }{n}},\,{z}_{{\rm{A}}0}=0,\end{array}$$

and *i* = 1, 2, ..., (*m* − 1)/2.

It is obvious from equations (19–21) that *ε* is dependent on *m*, and therefore *θ*_t_/*ε* is tuneable by both *m* and *n*. First consider the effects of *n* by taking *m* = 3 and *n* from 4 to 40. The relationship between *ε* and *n* is presented in Fig. 5b. The change tendency of *ε* is similar as *θ*_t_*vs*. *n*, but it varies more rapidly. As a result, except for the special case *n* = 4, *θ*_t_/*ε* is in general increased with the increase in *n* as shown in Fig. 5c, which shows a completely different trend from *θ*_t_. A minimum of *θ*_t_/*ε* = 5.8°/% is obtained when *n* = 5, which is almost triple of the maximum one in reference^31^.

The correlation between *θ*_t_/*ε* and *m*, is less clear, as can be seen in Fig. 5d in which *n* is fixed to 6. In this case the twist angle remains constant as 60° whereas the axial strain is changed with *m*, leading to the variation of *θ*_t_/*ε*. A maximum of *θ*_t_/*ε* = 37.2°/% is obtained when *m* = 7. Therefore, we can design mechanical metamaterials with a wide range of twist angle per axial strain by fine-tuning the geometrical parameters *m* and *n*. And such twist can be materialized with minimum efforts as it is a purely rigid motion.

### Non-rigid twist of the waterbomb tube

**Figure 6:**
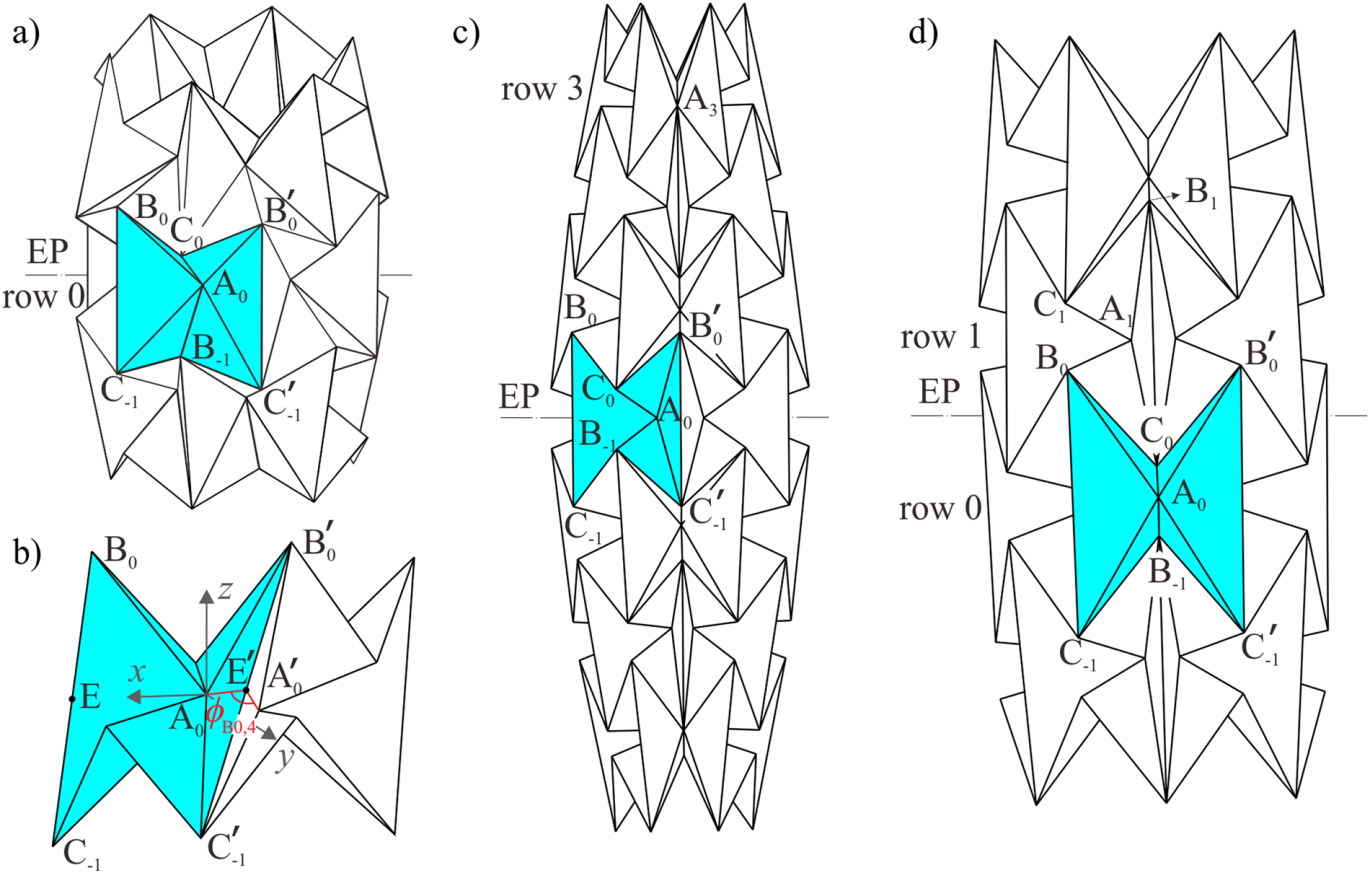
Non-rigid twist of the waterbomb tube when *n* = 6. (**a**) 3D view of a waterbomb tube with *m* = 3 when twist starts from the not-fully-squeezed line- and plane- symmetric row 0 with *ϕ*_B0,4_ ≠ 0. (**b**) Geometry of two adjacent bases on such not-fully-squeezed row 0. (**c**) 3D view of a waterbomb tube with *m* = 7 where the row 3 is fully squeezed with only plane symmetry. (**d**) 3D view of a waterbomb tube when twist starts from a pair of rows, set as row 0 and row 1. Only the twisted rows and those immediately adjacent to them are presented. EP is short for equatorial plane.

The sufficient condition of the rigid twist motion has been proved to be that the twisted row is fully squeezed with both line and plane symmetry. Now we are going to check its necessity. Firstly, we need to figure out whether the rigid twist motion will start if the line- and plane- symmetric spherical 6*R* linkage **A**_0_ is not fully squeezed, that is, *ϕ*_B0,4_ ≠ 0, see Fig. 6a. Two adjacent bases on row 0 of such a waterbomb tube is presented in Fig. 6b, where the coordinate system is the same as that in Fig. 3b. According to the spatial analytical geometry, the angle between the crease B′_0_C′_−1_ and the axis *z*, *γ*, can be calculated

22 $$\cos \,\gamma =\frac{1}{a}\sqrt{\frac{-{y}_{{\rm{B}}^{\prime} 0}^{2}+2a{y}_{{\rm{B}}^{\prime} 0}\,\sin \,\angle {{\rm{EA}}}_{{\rm{0}}}{\rm{E}}^{\prime} -{a}^{2}}{{\cos }^{2}\angle {{\rm{EA}}}_{{\rm{0}}}{\rm{E}}^{\prime} }+2{a}^{2}}.$$

As both planes A_0_E′A′_0_ and EA_0_E′ are perpendicular to crease B′_0_C′_−1_ and the axis *z*, respectively, the angle between the two planes EA_0_E′ and A_0_E′A′_0_ is also *γ*. Therefore the vertical distance between the vertices A′_0_ and A_0_ is

23 $${z}_{{\rm{A}}^{\prime} 0}-{z}_{{\rm{A}}0}=-\,\overline{{{\rm{A}}}_{{\rm{0}}}{{\rm{A}}^{\prime} }_{0}}\,\sin \,\gamma =-\,2a\sin \,\frac{{\varphi }_{\mathrm{B0},4}}{2}\,\sin \,\gamma .$$

If *ϕ*_B0,4_ ≠ 0, *z*_A′0_ − *z*_A0_ ≠ 0. According to the recursion formula in equation (23), the vertical distance between vertex A′_0_ and plane *x*A_0_*y* increases with the number of bases on row 0, which makes the vertex C′_−1_ of the *n*th base that is obtained after twist not match the vertex C_−1_ of the first base, so that the bases on row 0 cannot complete a cylindrical tessellation. Therefore, no rigid twist motion occurs when the line- and plane- symmetric row of the tube is not fully squeezed. To this point, we can conclude that only the twist of the fully squeezed row in the middle of the tube in Fig. 1b is a rigid motion.

Secondly, the necessity of line and plane symmetry is studied, that is, whether the twist motion is rigid if the twisted row is fully squeezed without line and plane symmetry. Figure 6c shows such a case that the row 3 is fully squeezed with only plane symmetry. Due to the lack of two-fold symmetry necessary to reach the bifurcation configuration, the plane-symmetric linkage **A**_3_ cannot bifurcate to a tilting motion. In other words, the twist motion on the fully squeezed row without both line and plane symmetry is not rigid.

Therefore, both the fully squeezed configuration and the line and plane symmetry are necessary for a rigid twist motion. Should either one be violated, the twist motion requires material deformation. Obviously, the twist motion with neither fully squeezed configuration nor line and plane symmetry is not rigid. There are two cases of such non-rigid twist motion. First, when the twist occurs on the fully squeezed row 0, the bases on the other rows is only plane-symmetric and not fully squeezed, so the successive twist of other rows after row 0 reaches its limit positions (Fig. 4a,b) is non-rigid and it cannot occur without material deformation. Second, when the twist motion occurs from a pair of rows near the equatorial plane, which are set as rows 0 and 1 as shown in Fig. 6d, the bases on all rows are not fully squeezed and have only plane symmetry. As a result, there is no rigid twist motion. However, playing with the physical model shows that twist exists in this case as well, and such a process is transmitted from row to row towards the ends of the tube, see Supplementary Videos S2 and S3. So we can safely conclude that, the entire twist motion is due to material deformation. Notice that some rows twist clockwise while the others twist counter-clockwise. The reason is that in such a way, the relative rotation of the two ends of the tube can be cancelled out.

**Figure 7:**
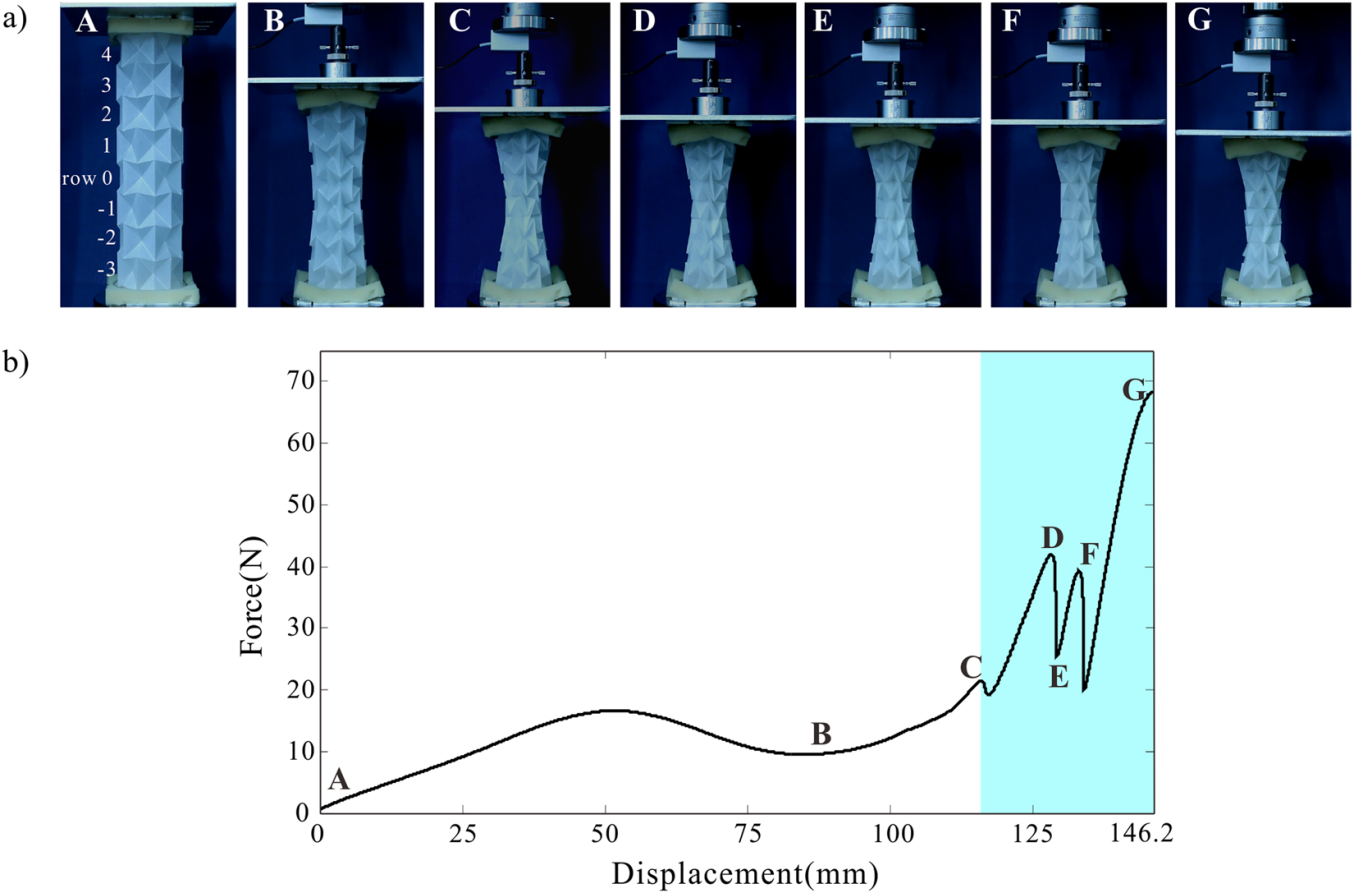
Axial compression experiment of the waterbomb tube. (**a**) Compression process of the tube. (**b**) Reaction force of the tube *vs*. axial displacement curve. The tube in the experiment took a uniform radius with the following geometric parameters: *n* = 6, *m* = 8, *a* = 22.5 mm, and initial dihedral angle *θ* = 144°. ENDURO Ice material with 0.29 mm in thickness was used to construct the tube. The compression test was conducted on an Instron machine at the loading rate of 5 mm/min.

The discovery of the twist motion enables design of origami structures and mechanical metamaterials with graded stiffness through a combination of contraction and twist. Such behaviour is demonstrated by a quasi-static axial compression of a waterbomb tube with *n* = 6 and *m* = 8. As can be seen in Fig. 7a, a radial contraction occurs at the beginning of the compression, with a larger shrinkage in the middle than both ends due to boundary constraints, see configuration B. The contraction phase ceases when row 0 and row 1 are fully contracted in configuration C, followed by a simultaneous twist of both rows in opposite directions as seen in configuration D. It is known from the analysis above that the twist is structural deformation instead rigid motion. The twist phase proceeds as row 2 and row −1 twist successively (configurations E and F), after which local material damages appear and the experiment is terminated (configuration G). Regarding stiffness, the force *vs*. displacement curve in Fig. 7b indicates that the force is low during the contraction phase before configuration C. With the occurrence of twist, the force level is raised significantly as shown in the shaded region of Fig. 7b, which demonstrates a periodic manner corresponding to the successive twist motion. The local peaks in the twist stage are approximately doubled in comparison with that in the contraction stage. Such graded stiffness would enable the structure/metamaterial to autonomously adapt to non-uniform loading environment. And this adaption is achieved purely through a structural transition of deformation phase, without requirement of gradation in the geometric or material dimensions.

## Conclusions

We have disclosed and explained the nature of the twist motion of the waterbomb tube that follows the commonly known contraction motion. Through a detailed kinematic analysis, the sufficient and necessary condition of a rigid twist motion has been revealed at the fully squeezed line- and plane- symmetric row in the end of contraction. The rigid twist motion range has also been determined, which is related to both the left/right handed twist and the most expanded configuration at the end rows. The twist angle per axial strain of the waterbomb tube with rigid twist motion has been analysed, which generally increases with the number of bases in a row. In addition, the behaviours of non-rigid twist motions have been studied. The significant difference in stiffness of the waterbomb tube with and without twist has also been verified by experiments. These new findings make the waterbomb tube ideal for the design of programmable and tuneable mechanical metamaterials.

## Methods

The card model shown in Fig. 1b was made from conventional cards obtained from stationary stores. The prototype shown in the Supplementary Video S2 was made from an ENDURO Ice sheet of 0.29 mm in thickness. The prototype shown in the Supplementary Video S3 was fabricated as a complete structure by a 3D printing machine OBJET Connex 350^©^ using two types of materials: a hard plastic-like one known as Verowhite^©^ for the facets and a soft rubber-like one called Tangoblack+ ^©^ for the creases, resulting in the facets being much stiffer than the creases. The prototype had *n* = 6, *m* = 8, *a* = 23 mm, wall thickness *t* = 1 mm, and an initial dihedral angle *θ* = 144°, where *θ* is the angle between the two largest triangle triangular facets of a base on row 0.

To demonstrate the graded stiffness of the waterbomb tube, a tube made from ENDURO Ice material with 0.29 mm in thickness and *m* = 8, was compressed in the longitudinal direction from the larger uniform radius configuration with an initial dihedral angle *θ* = 144°. It has the following geometrical parameters: *n* = 6 and *a* = 22.5 mm. The experiment was conducted on an Instron 5982 testing machine with a load cell of 100 N. The loading speed was chosen as 5 mm/min so that material strain rate effects could be safely neglected. Regarding boundary conditions, it was determined after several rounds of trial-and-errors that placing foams of 15 mm in thickness at each end of the tube, as shown in Fig. 7a, was able to generate a roughly symmetric and stable deformation.

## Electronic supplementary material

Supplementary Information 

Video S1

Video S2

Video S3
