# Supplementary material for: Twist of Tubular Mechanical Metamaterials Based on Waterbomb Origami
Source: Sci Rep. 2018 Jun 22;8:9522. doi: 10.1038/s41598-018-27877-1 (PMC6015070; doi:10.1038/s41598-018-27877-1)
Supplement: Supplementary file 1 — Supplementary Information [file 41598_2018_27877_MOESM1_ESM.docx]

**Twist of Tubular Mechanical Metamaterials Based on Waterbomb Origami**

Authors: Huijuan Feng^1, 2^, Jiayao Ma^1, 2^, Yan Chen^1, 2*^, Zhong You^3^

Address:

^1^ Key Laboratory of Mechanism Theory and Equipment Design of Ministry of Education, Tianjin University, Tianjin 300072, China

^2^ School of Mechanical Engineering, Tianjin University, Tianjin 300072, China

^3^ Department of Engineering Science, University of Oxford, Parks Road, Oxford, OX1 3PJ, UK

* Corresponding Author <yan_chen@tju.edu.cn >

**Supplementary information**

Videos:

1. Video S1: animation of the rigid twist motion of the waterbomb tube with $n=6$, and $m=3$ in Figure 3;
2. Video S2: twist motion of a waterbomb tube made from an ENDURO Ice sheet which occurs from a pair of rows near the equatorial plane; and
3. Video S3: twist motion of such waterbomb tube made by 3D printing.
